# Biophotonic approach for the characterization of initial bitter-rot progression on apple specimens using optical coherence tomography assessments

**DOI:** 10.1038/s41598-018-33791-3

**Published:** 2018-10-25

**Authors:** Ruchire Eranga Wijesinghe, Seung-Yeol Lee, Naresh Kumar Ravichandran, Muhammad Faizan Shirazi, Pilun Kim, Hee-Young Jung, Mansik Jeon, Jeehyun Kim

**Affiliations:** 1 School of Electronics Engineering, College of IT Engineering, Kyungpook National University, 80 Daehak-ro, Buk-gu, Daegu, 41566 Korea; 2 School of Applied Biosciences, Kyungpook National University, 80 Daehak-ro, Buk-gu, Daegu, 41566 Korea; 3 Department of Biomedical Engineering, College of Engineering, Kyungil University, 50, Gamasil-gil, Hayang-eup, Gyeongsan-si, Gyeongsangbuk-do, 38428 Republic of Korea; 4 Center for Medical Physics and Biomedical Engineering, Medical University of Vienna, Waehringer Guertel 18–20, A-1090 Vienna, Austria; 5 Institute of Biomedical Engineering, School of Medicine, Kyungpook National University, 680, Gukchaebosang-ro, Jung-gu, Daegu, 41944 Korea

## Abstract

The tremendous advances achieved in the biophotonics technologies have intensified the necessity for non-invasive modalities that can characterize diverse biological materials with increased sensitivity and resolution. Optical coherence tomography (OCT) is one of the techniques that has been applied for biological applications in medicine and agriculture to identify structural properties. Herein, we report the successful incorporation of OCT for the identification of morphological changes that occur as a result of the bitter rot disease, through continuous detection of structural changes. Detailed inner morphological structural changes occurring in fruit specimens were precisely analyzed as a function of the disease incubation period using OCT. The conducted histological correlation and quantitative three-dimensional evaluations provide a robust platform for further discoveries related to plant materials. The results highlight the initial identification of bitter rot progression on apple specimens owing to the non-invasive inspection capability of OCT. Therefore, we expect that the proposed method will enable immediate sensitivity improvements in the inspection of plant diseases for postharvest utility.

## Introduction

Bitter rot caused by *Colletotrichum spp*. is one of the destructive diseases in apple cultivations^1,2^. The initial symptoms include the appearance of small gray or brown spots, typically encountered as circular spots that expand from the center of the infected region. Furthermore, this disease is known as a postharvest disease and frequently occurs during fruit storage^2^. An effective fungicide spray program in the field and appropriate postharvest storage conditions are essential for controlling apple bitter rot disease. The identification of the initial symptoms of this disease is essential and has not been studied descriptively. Hence, accurate non-contact strategies are being explored by the research community to circumvent these issues.

Histological analysis and the polymerase chain reaction (PCR) technique have been frequently utilized as gold standard methods to detect plant related diseases^3–8^. However, these time consuming and destructive experimental procedures affect the biological nature of specimens, and ultimately reduce the experimental accuracy. In fact, most extensively investigated non-destructive plant related disease inspection techniques, such as visual inspection, magnetic resonance imaging (MRI), X-rays, positron emission tomography (PET), confocal microscopy, fluorescence spectroscopy, and near infra-red (NIR) spectroscopy, have been implemented by numerous research groups^9–16^. However, low resolution, long acquisition times, and limited depth penetration, are major drawbacks of these methods, which ultimately limit their applications in agriculture.

Characterization of the aforementioned initial stages of bitter rot infection can only be achieved using a non-destructive inspection technique that provides exceptional morphological information with high-resolution. Thus, to explore these initial symptoms at a micrometer resolution, optical coherence tomography (OCT) constitutes a successful approach that can lead to vast improvements in imaging sensitivity and produce much stronger optical signals^17–21^. OCT represents a promising diagnostic approach for numerous plant related research studies, since researchers have already employed it for the quantitative analyses of seeds^22,23^ and the inspection of various plant diseases^24–27^. Furthermore, OCT has emerged as a non-destructive technique to assess peel structure properties of apple and kiwi fruit specimens for the investigation of structural property changes that occur owing to fertilization treatments and storage duration^28–30^. Hence, OCT has become one of the mainstream diagnostic options owing to the growing interest in the field of biology and agriculture. For initial identification, accurate localization, and precise characterization of plant diseases, our group has recently developed several OCT-based inspection methodologies to detect well known plant diseases^31–37^. Furthermore, we established a bi-directional scanning method to enhance the depth visibility of plant specimens^38^. Most importantly, a wearable-OCT (backpack-type) system was developed by our group to functionalize OCT as an *in situ* inspection method^39^.

To validate the non-contact and micrometer resolution benefits of OCT, we demonstrate herein an extended agricultural application by synthesizing a 1310 nm swept source OCT (SS–OCT) system to characterize the initial symptoms of apple bitter rot disease. The morphology of the studied specimens was examined longitudinally over periods of 25 days (25 d) as a function of depth and structural changes along the lateral direction. The acquired results were quantitatively investigated based on optical signals and boundary detection techniques. According to our knowledge, inner morphological changes in apples caused by bitter rot disease have not been previously studied in plant biology in two dimensions (2D), three dimensions (3D), or quantitatively, using non-destructive methods or OCT to this date. Thus, the structural and quantitative evaluations successfully conducted herein sufficiently confirmed the initial stages and further growth of bitter rot disease, which could help the development of appropriate prevention methods for postharvest utility. The results of the histological analysis that were performed simultaneously, were closely correlated with these evaluations, and confirmed a similar behavior to that observed in the acquired 2D OCT images.

## Materials and Method

### OCT system description

**Figure 1:**
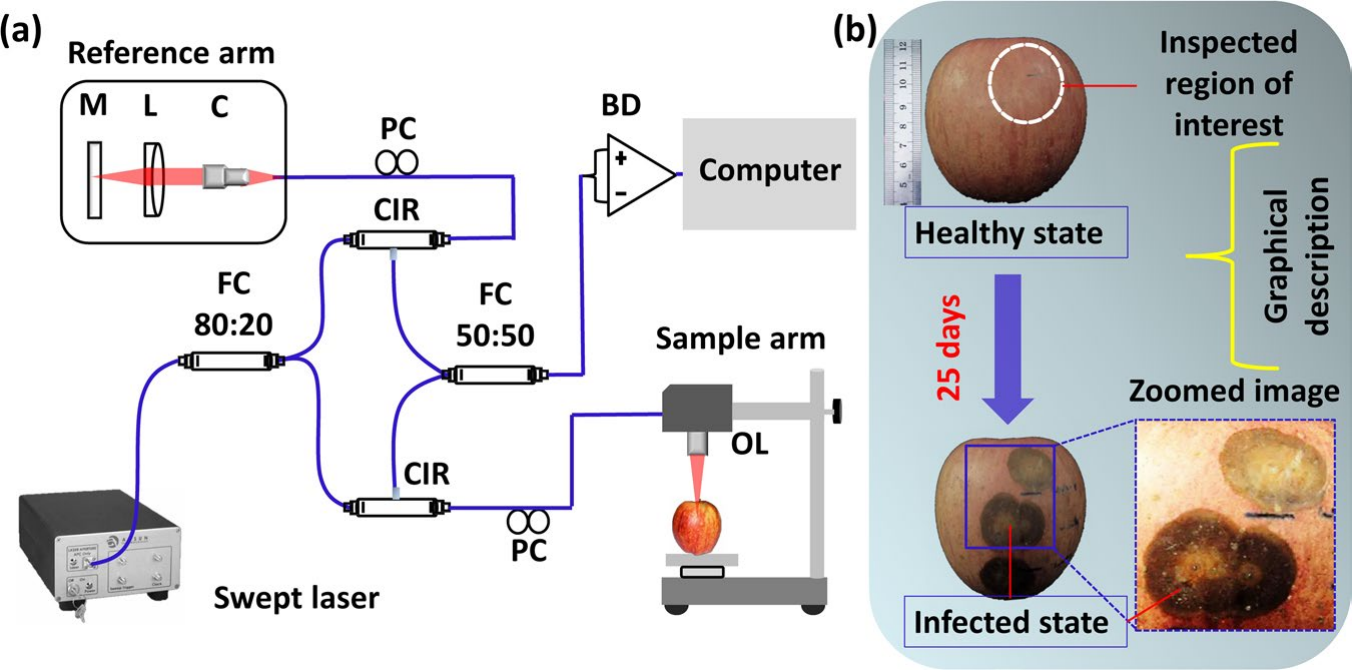
(**a**) Schematic diagram of the SS-OCT system configuration. (**b**) Graphical representation of the inspected fruit specimen. Abbreviations: BD, balanced detector; C, collimator; CIR, circulator; FC, fiber coupler; L, lens; M, mirror; OL, objective lens; PC, polarization controller.

The customized SS–OCT system configuration is schematically presented in Fig. 1(a). The utilized commercial swept source was a 1310 nm central wavelength swept laser (Axsun Technologies, USA), with 12 mm coherence length, 110 nm sweep bandwidth, average output power of 20 mW, and a sweeping rate of 100 kHz. Light from the laser source is conveyed towards the sample and reference arms via the optical fiber coupler (Gooch & Housego, UK) at a splitting ratio of 50:50. The two output terminals of the fiber coupler were connected to a balanced photodetector (Thorlabs, USA). The acquired output signal at the balanced photodetector was digitized using a 12-bit waveform digitizer (Alazar Technologies Inc., Canada). Image construction was performed using a software-based data processing method. The specimens were continually examined at a scanning range of 4 mm × 4 mm, and the applied refractive index was 1.42^40^. The measured axial resolution of the system was 7.5 μm (in air) and the lateral resolutions were 10 μm (in air) and 7 μm (in plant tissues). The detailed configurations of the OCT instrumentation are provided elsewhere^23^. The graphical description of the region of interest of apple specimens (photographs captured at the laboratory) and the monitoring procedure is emphasized in Fig. 1(b).

### Cross-sectional OCT-image based quantitative evaluation procedure

**Figure 2:**
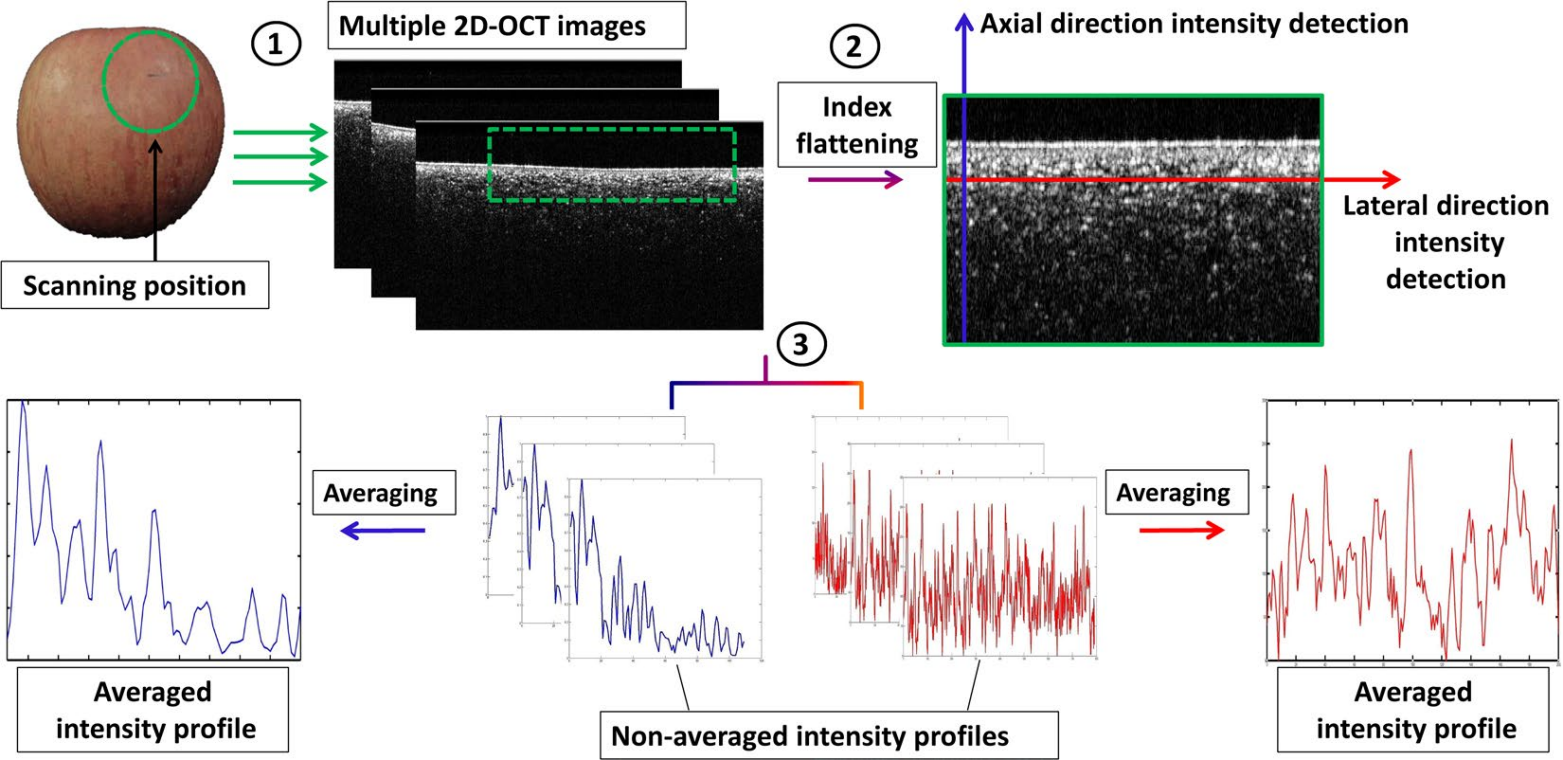
Graphical explanation of the OCT intensity detection algorithm along the axial and lateral directions.

The precise analysis of the OCT signal fluctuation along the axial (depth) and lateral directions constitutes a structural refractive index-based evaluation procedure, since the refractive index of each tissue component is unique and differs from other tissues. Although each component of the tissue has a unique refractive index, herein, we used a refractive index equal to 1.42, which is the fundamental refractive index of plant cells. Therefore, once the particular refractive index is applied to the cross-sectional images, the corresponding axial direction depth profiles (depth A-scans) and transverse direction lateral intensity profiles (lateral A-scans) of the cross-sectional image provide information about the inner morphology in both the axial and lateral directions in association with thickness measurements. A custom-made MATLAB (Mathworks, USA) program was developed for axial and lateral direction A-scan profile analyses. The 2D OCT images were loaded into the program and a peak search algorithm based on an image window with 300 intensity signals (A-scans) was applied to analyze the A-scans. The program was simulated separately for axial and lateral intensity signals. The algorithm detected the maximum intensity in each individual A-scan line sequentially. All the maximum intensity positions in all 300 A-scan lines were then rearranged, linearly indexed (to flatten the region of interest), summed up, averaged, and normalized to acquire (axial and lateral) A-scan profiles, as indicated in Fig. 2. A detailed account of the algorithm is beyond the scope of this study. Instead, we refer readers to several comprehensive literature reports that provide sufficient knowledge^34^.

**Figure 3:**
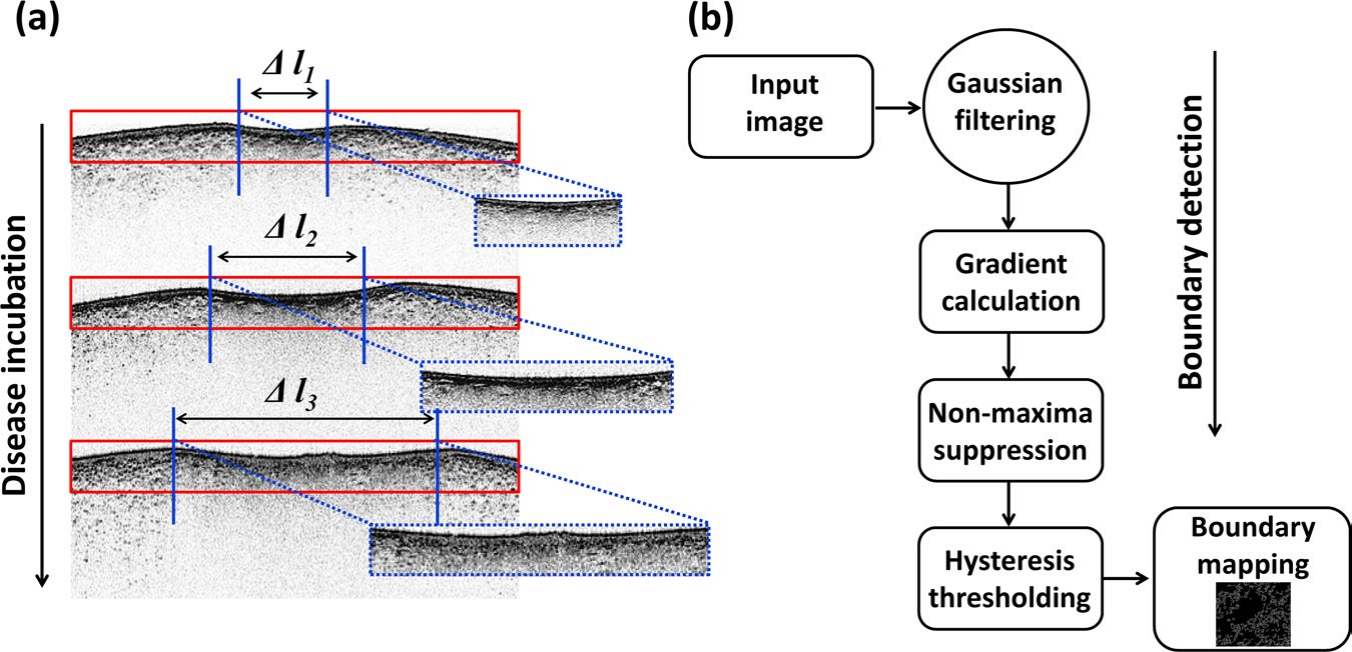
(**a**) The representation of the lateral direction expansion of the infected region as a function of the period of incubation. (**b**) Flow chart representing the main steps of the tissue boundary detection method. (*Δl*_1_ *~ Δl*_3_ illustrates the lateral direction expansion of the infected region).

In addition to axial OCT signal analyses, gradual expansion of the lateral direction thickness of the infected areas in the epidermis and hypodermis regions were characterized as a function of the incubation of the disease. Hence, the aforementioned algorithm was implemented for a better understanding and a rigorous confirmation of the gradual thickness expansion along the lateral direction. The thickness was estimated by considering the distance between average peak information of the lateral A-scans. Figure 3a illustrates the region-of-interest of specimens and the expansion of thickness. To increase the accuracy of the measurements, six distrustful regions from a single fruit specimen were selected and multiple 2D OCT images (ten images) were acquired from each distrustful region, and the average value of the measurements was used. We then quantified the thickness expansion for all the specimens and calculated the average value to gain more precise thickness information. Additionally, a boundary detection algorithm (Canny algorithm) was utilized to extract the boundary information of infected tissue regions in enface OCT images that confirmed the deep morphological structural differences between healthy and infected samples. Since this technique localizes partially infected tissue boundaries, the most important region-of-interest can be determined and non-useful information can be discarded. The flow chart shown in Fig. 3b depicts the main stages of the boundary detection algorithm followed by the application of the methods reported in^41,42^.

### Histological procedure

Apart from the OCT-based continuous non-destructive inspection process, an additional set of specimens was selected for sequential histological inspections from the same plantation. Histology sections were prepared in a standardized style for comparison with 2D OCT images. These had to be matched to the OCT recording axis. To obtain a precise correlation with the acquired 2D OCT images, the selected specimens were fixed in 2% paraformaldehyde and 2.5% glutaraldehyde in 0.05 M sodium cacodylate buffer for 24 h under vacuum conditions. Subsequently, the specimens were rinsed with distilled water and dehydrated serially in graded ethanol solutions using 30%, 50%, 70%, 80%, and 90% absolute ethanol for periods of 20 min. The dehydrated specimens were infiltrated with propylene oxide and embedded in Spurr’s resin. Finally, the samples were polymerized in 100% Spurr’s resin at 70 °C for 8 h. After polymerization, 2 µm thin sections were cut using an ultra-microtome (MT–7000, RMC, Tucson, AZ, USA), stained with 2% methylene blue, and observed under a light microscope (BX50, Olympus, Tokyo, Japan).

### Fruit specimen preparation

Fruit specimens for OCT-based continuous structural and histological examination were collected from naturally infected apple orchards located in Korea three weeks after the harvest. Healthy and apparently bitter rot infected fruit specimens were collected from 10 uniform trees. To maintain the biological nature of the fruit structure, the specimens were stored in a temperature-controlled room at temperatures of 15 ± 0.5 °C during the entire 25 days monitoring period. Among the fruit specimens, 80% of them were inspected using OCT and the remaining 20% underwent histological examination. All plant material manipulation procedures had been approved by the Applied Bioscience Research Ethics Committee of Kyungpook National University.

## Results

### Continuously inspected structural variations and thickness analysis

**Figure 4:**
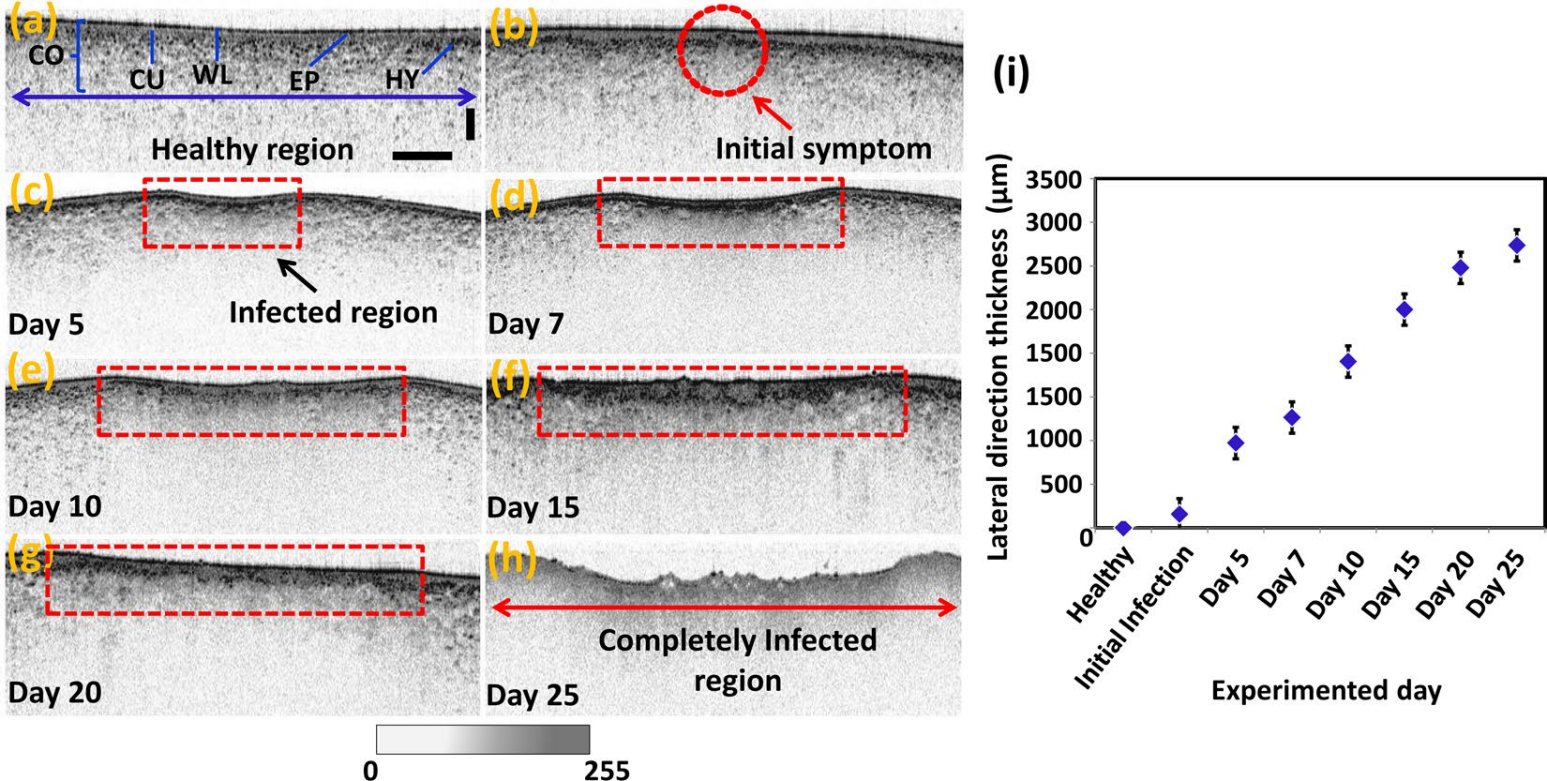
Morphological changes as a function of the incubation of bitter rot disease (**a–h**). Graph (**i**) depicts the gradual thickness expansion of the infected region in the lateral direction. Abbreviations: CO, cortex; CU, cuticle; EP, epidermis layer; HY, hypodermis layer; WL, wax layer. Vertical and horizontal scale bars, 500 μm.

Our OCT study enabled continuous observation of morphological changes in apples caused by bitter rot disease following incubation. The specimens, which were either healthy, or apparently healthy but infected, were examined for 25 consecutive days. The internal layers were well differentiated at the healthy stage, as shown in Fig. 4a. Importantly, clear structural destructions in wax and epidermal layers of apparently healthy but infected specimens were observed in OCT images, as shown in Fig. 4b. We further explored a gradual increase of the infected region in lateral direction and gradual disappearance of the cuticle, wax, epidermis, and hypodermal layers in the axial direction during the 25 days of consecutive monitoring (Fig. 4c–h). These OCT images contain red-color dashed regions characterizing the regions-of-interest in all specimens revealing the expansion of the infected areas. Therefore, it can be emphasized that the high contrast sensitivity and micrometer resolution of OCT can be advantageous to detect bitter rot disease at an initial stage compared to conventional techniques.

Furthermore, we quantified and compared the gradual thickness expansion of the infected region in lateral direction as a function of the progress of bitter rot disease. The initial selection of the exact location of infection was a challenging task. Therefore, six distrustful locations were randomly selected and continually monitored in each fruit specimen. Among the selected locations, the regions, which demonstrated the abnormal morphological behavior due to rotting were selected for the quantification analysis. Multiple sets of ten 2D OCT scans were approximately acquired from regions close to the aforementioned distrustful locations, thereby enabling the matching of the positions of the particular 2D OCT images with the images acquired on previous experimental days. Gray-scale pixel measurements based on the lateral thickness variation were evaluated using customized software to analyze the thickness expansion. To increase the accuracy of the pixel detection method and to determine the exact thickness, measurements were obtained from the entire set of the 2D OCT scans (ten 2D OCT scan sets acquired at each location), and the measurements were averaged. We then analyzed the thickness expansion for all 80 fruit specimens and averaged them to obtain precise evidence regarding the expansion of the thickness as a function of the bitter rot incubation. As expected, based on the acquired cross-sectional images, a gradual increase was confirmed in the lateral direction of the region-of-interest (shown in red-color dashed region). To gain a clear understanding, the obtained statistical values and quantified outcomes, such as the minimum, maximum, averages, and standard deviations estimated for each observation day, are listed in Table 1, and the graphical representation is illustrated in Fig. 4i.

**Table 1:** Statistical data of thickness expansion along the lateral direction.

State of specimens	No. of specimens	Minimum thickness (µm)	Maximum thickness (µm)	Average thickness (µm)	Standard deviation
Healthy	80	—	—	—	—
Initial state	80	154.42	168.84	160.88	±3.35
After 5 days	80	925.16	987.54	968.86	±13.92
After 7 days	80	1243.21	1288.15	1263.04	±9.37
After 10 days	80	1904.41	2043.58	1989.32	±12.97
After 15 days	80	2500.24	2572.52	2505.22	±14.81
After 20 days	80	2965.11	3011.78	2977.92	±8.99
After 25 days	80	3234.81	3334.69	3234.88	±13.85

### Histologically correlated cross-sectional analysis

**Figure 5:**
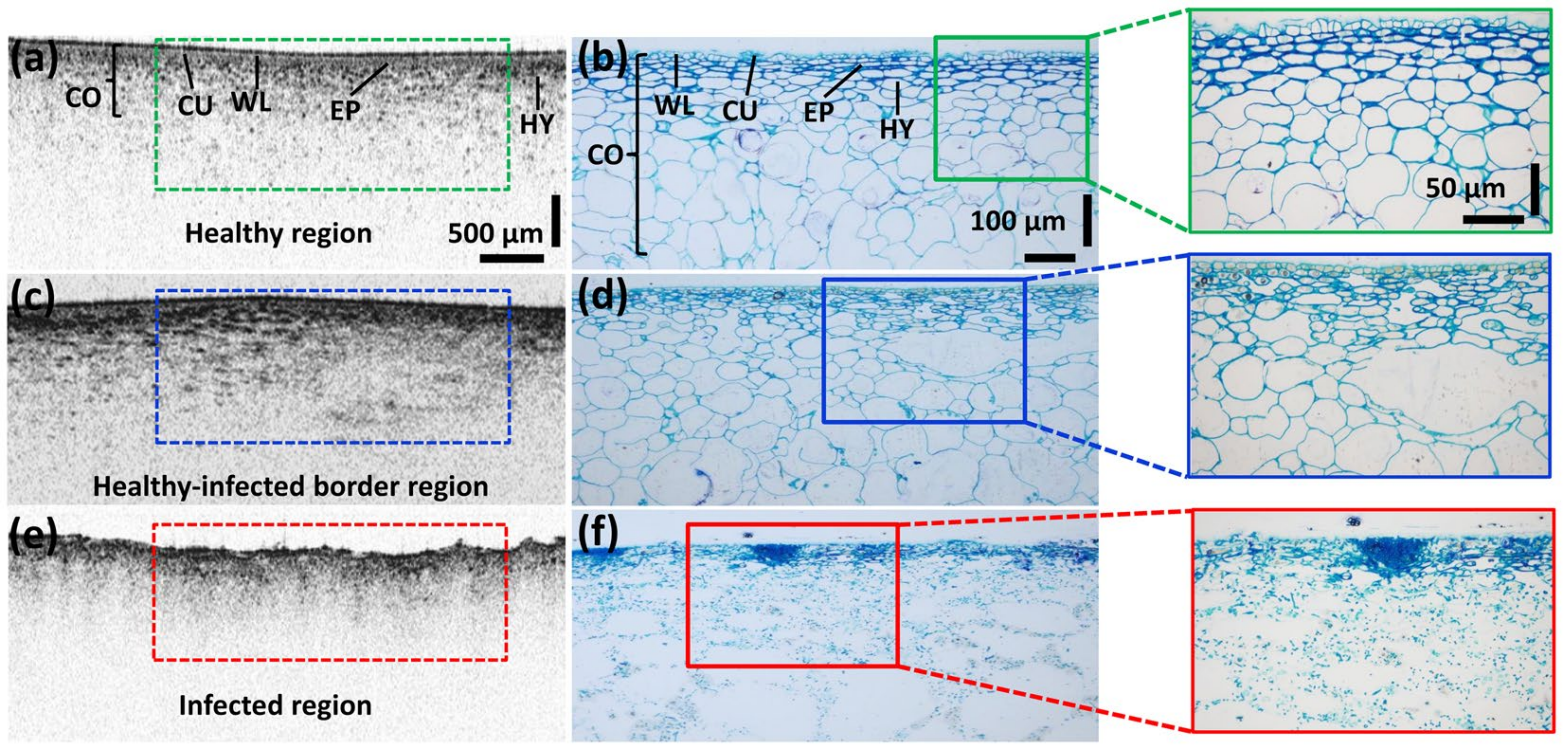
Histological confirmation of the 2D OCT images acquired from healthy and naturally infected fruit specimens. (**a,b**) Images representing the morphology of healthy specimens. (**c,d**) Images representing the cross-sections of a partially infected (apparently healthy) specimen. Finally, (**e,f**) represent the cross-sections of an entirely infected specimen. The zoomed version of each histological section is shown in the right corner. Abbreviations: CO, cortex; CU, cuticle; EP, epidermis layer; HY, hypodermis layer; WL, wax layer.

Knowledge of existing correlations between the OCT and histological analysis is important in related OCT studies of plant diseases. In this study, the presence and absence of inner morphological structures observed during the continuous 2D OCT observation process were confirmed and were found to be correlated with histologically sectioned images. Figure 5 shows matched histological–OCT pairs acquired from approximately identical scan levels. In healthy specimens, inner morphological structures, such as wax layers, cuticles, epidermis and hypodermis layers (along with the magnified histological images), were clearly evident in the OCT images (Fig. 5a,b). However, the disappearance of the aforementioned layers and morphological boundaries were observed from both histological and OCT observations of healthy appearing but infected (apparently healthy) specimens, as shown in Fig. 5c and d. Similarly, complete disappearance of all the layers and the formation of a consolidated single thick layer were confirmed in the histological observations of the infected regions, thereby emphasizing the similar behavior to the examined cross-sectional OCT images (Fig. 5e,f). Despite these precise correlations, the plant cell wall boundaries were barely visible in the OCT images owing to the limited depth penetration and resolution, which limited the ability to characterize them.

### Three-dimensional analyses of enface structural changes along the depth direction

**Figure 6:**
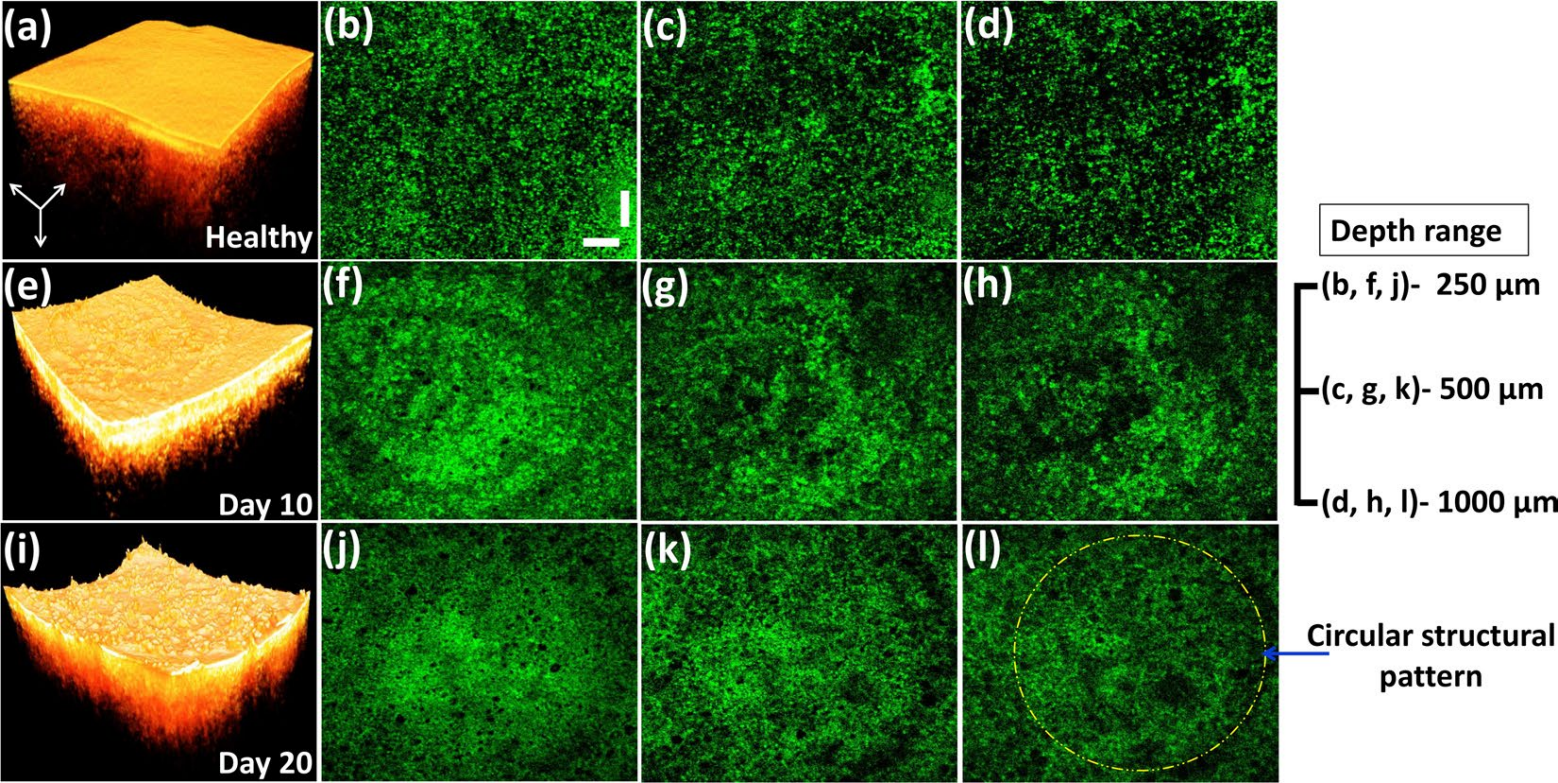
Illustration of the structures of a healthy specimen along the depth direction and structural differences according to the progress of the disease infection. The enface images were obtained from the corresponding depth levels of 250 μm, 500 μm, and 1000 μm, from each specimen. The dashed yellow-colored circle depicts the formation and the spread of circular tissue patterns in infected regions. Vertical and horizontal scale bars, 500 μm.

The three-dimensional analyses of the depth direction enface OCT images well below the tissue surface are highlighted in Fig. 6, illustrating sequential enface images acquired at depths ranging between 250 μm, 500 μm, and 1000 μm, respectively. Particularly, in healthy specimens, the distinguishable random tissue distribution can be well visualized in all depth ranges, as evident in Fig. 6b–d. Nevertheless, owing to the major initial symptoms of bitter rot disease, namely, the appearance of the circular spots that reside below the epidermis layer, dissemination of partially merged circular tissue patterns and boundaries can be screened in all three depth ranges, as shown in Fig. 6f–h. Subsequently, the extended spots, which develop in the center of the infected region and lead to an appearance of a bullseye boundary, can be explored on the 20th experimental day, as depicted in Fig. 6j–i. These distinguishable tissue distribution patterns localized in depth directions cannot be pre-identified using conventional agricultural inspection techniques. Therefore, the exploitation of non-contact inspection benefits of OCT can be taken into account for the initial assessment of the growth of bitter rot disease in apple specimens.

**Figure 7:**
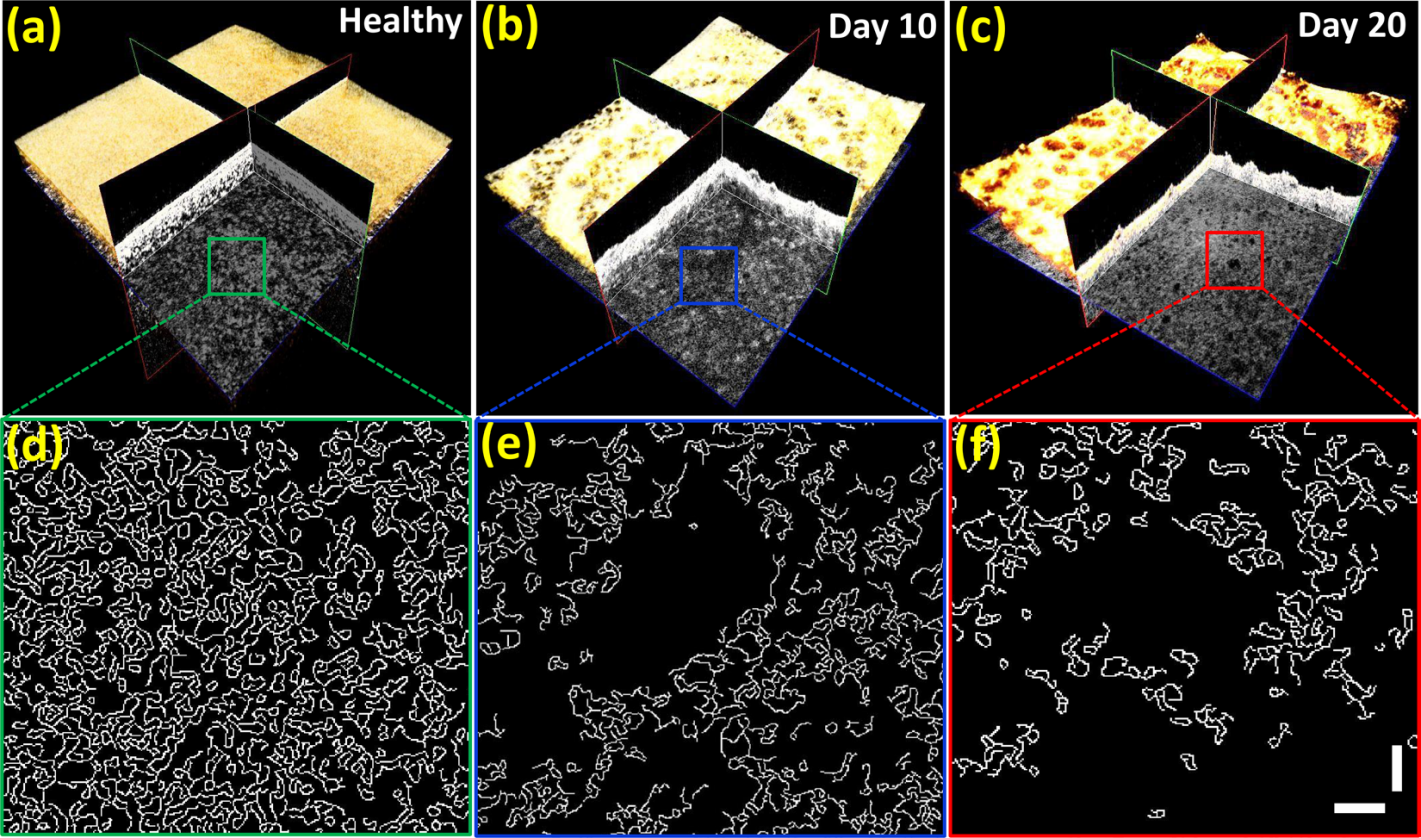
Tissue boundaries and behavioral changes of tissue patterns with respect to the progression of the disease using a boundary detection technique. Vertical and horizontal scale bars, 100 μm.

To explain the tissue distribution differences observed between healthy specimens and infected specimens on day 10 and day 20 in detail, the presence of diverse tissue patterns was investigated using the boundary detection method described in Section 2.2. Figure 7 demonstrates the corresponding tissue boundaries and behavioral changes of tissue patterns with respect to the progression stage of the disease. The enface images in the depth direction (acquired at a depth range of 1000 μm), and the magnified corresponding boundary detected images of squared regions, are shown in Fig. 7 to provide a better visualization of the tissue walls.

**Figure 8:**
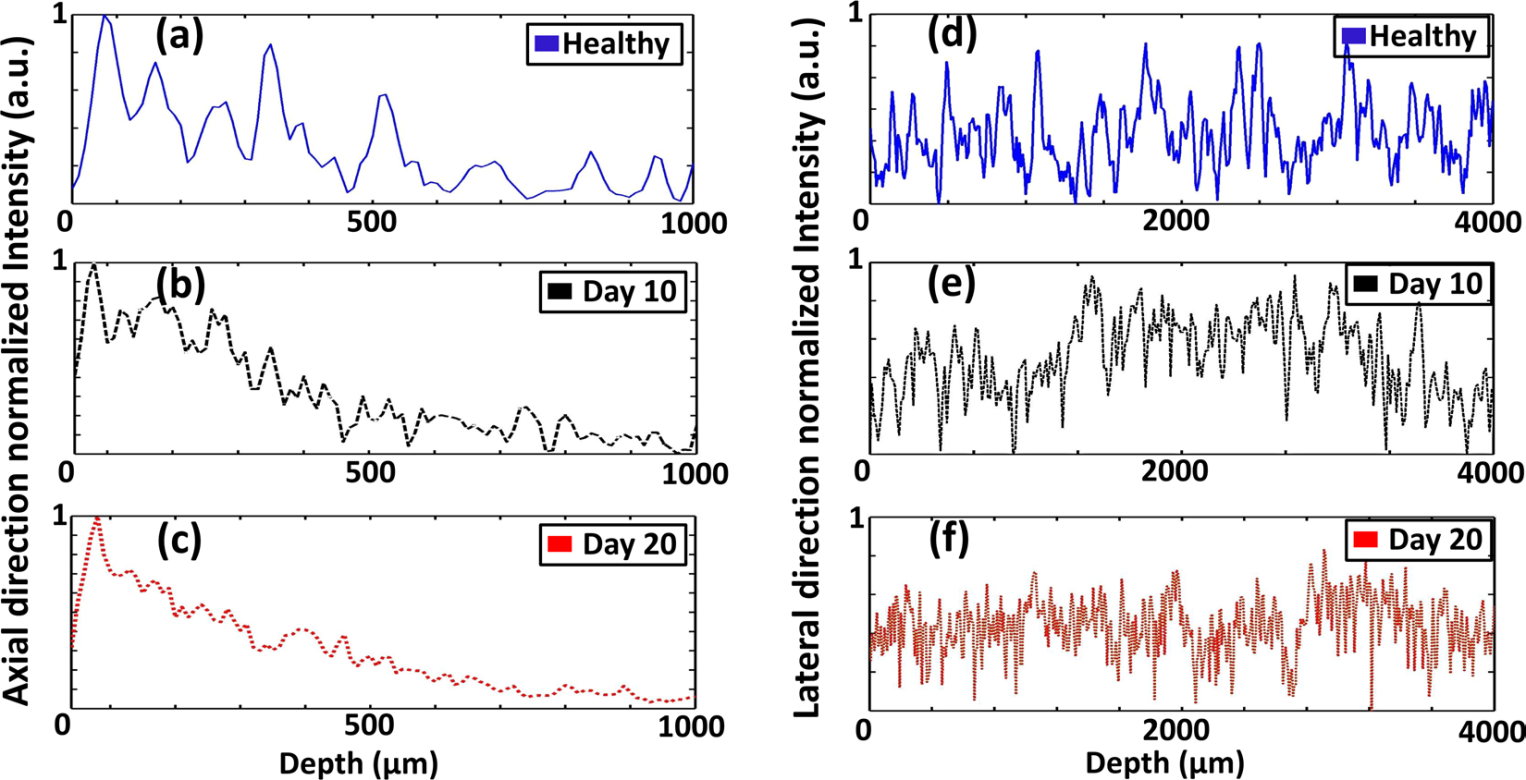
Normalized axial direction A-scan depth profiles and lateral direction intensity profiles evaluated from healthy specimens, and infected specimens respectively acquired at 10 and 20 days after the specimen collection.

To confirm morphological assessments of each assay, we analyzed the OCT signal fluctuations along the axial and lateral directions based on the acquired 2D OCT images. Therefore, successful cross-sectional images obtained from healthy and infected specimens on day 10 and day 20 after specimen collection were considered for this analysis. The normalized axial direction A-scan depth profiles are emphasized in Fig. 8a–c. Continuous and well distinguishable peaks represent the layer information (Fig. 8a presents data corresponding to wax, epidermis, hypodermal, and the other layers, respectively). As expected, based on the morphological assessments, the partial disappearance of the peaks was observed in A-scan profiles obtained on day 10, which correlates to the progression of the disease and confirms the initial symptoms. Thus, it is clear that the occurrence of initial symptoms leads to an internal destruction of layers. A similar analysis was applied to cross-sections obtained on day 20. A barely distinguishable single-peak representing the consolidated layer was detected in the A-scan profile, which reveals the disappearance of the entire spatial span of the internal layers following the incubation period. The same evaluation process was repeated to analyze the lateral direction intensity fluctuation, and the obtained results are shown in Fig. 8d–f. Herein, the analysis of the lateral intensity fluctuation was critical in observing detailed morphological characteristics owing to the expansion of the infected region. A similar signal behavior was observed to that elicited in the previous analysis, since clearly identifiable peaks (Fig. 8d), partially merged and barely distinguishable peaks (Fig. 8e), and entirely merged peaks (Fig. 8f) were detected on the cross-sectional images acquired from three corresponding specimens. Although the differences in the refractive indices play an important role in obtaining the scattering information from the internal layers, the fundamental refractive index of plant cells was applied to reveal the intensity profile differences between partially, entirely infected, and healthy specimens.

## Discussion

Purely non-destructive OCT can be considered as a growing mainstream agricultural inspection technique, which offers reasonable hope to address many devastating plant diseases, disorders, and plant-related vectors. In our previous studies, we successfully confirmed the non-destructive initial identification capability of various plant diseases using OCT^31–37^. Despite these previous applications, to-this-date, plant morphology in the context of initial symptoms of bitter rot disease in apples has not been investigated using non-destructive techniques. Hence, we evaluated the use of OCT in the analyses of the changes in morphology owing to bitter rot disease following incubation. The non-destructive inspection capability of OCT was well exploited for peel structural properties of apple specimens with a detail comparison between confocal microscopy and micro-CT images^29^. Although the resolution of confocal microscopy is higher than OCT, the identifiable volume and field of view is limited. Also, the usefulness of enface OCT representations for plant specimens was confirmed through the visualizations of internal tissue cracks. Moreover, the postharvest utility of OCT was verified through investigation of structural properties underneath the periderm of kiwi fruit specimens using qualitative 2D-OCT and 3D-OCT images^30^. Additionally, an automated image processing protocol was introduced to visualize larger near skin cellular structures in the outer parenchyma layer of kiwi fruit specimens. The reported method was fundamentally involved for the kiwi fruit quality inspection during the time of harvest and storage, which was helpful to distinguish large cells and stone cells. OCT was further applied to investigate ring breakdown of oil glands in “Nules Clemantine” (mandarin) fruit specimens providing qualitative histological and microstructural properties^25^. The automatic image analysis procedure was succeeded in shape extraction of oil glands, while directly providing guidance to the identification of ring breakdowns of oil glands. Based on these promising findings, the main conceptual and experimental objective of the study was focused towards the pre-identification of bitter-rot progression in apple specimens.

Numerous agricultural observation techniques have been reported for postharvest detection of bitter rot disease in apples^2,43,44^ where bitter rot symptoms were studied using mitochondrial (mt) DNA restriction fragment length polymorphism (RFLP) haplotypes and PCR examinations. The extraction of genomic DNA is an essential procedure, which hampers the structural properties of fruit specimens. Pre-identification of the presence of the initial rotting of apples is a crucial finding that can be rigorously succeeded with use of a high-resolution non-destructive inspection technique. However, prior research studies had mainly focused on the inhibition of apple rotting using spore and mycelial mat antagonists or suspensions of their crude extracts, and achieved bitter rot prevention through calcium treatments^45–47^.

The competency of the proposed OCT methodology to identify the initial bitter rot disease symptoms suggests the potential applicability of OCT to various agricultural applications and plant diseases. Although numerous destructive and non-destructive techniques have been implemented for plant disease inspection, OCT has considerable merits compared to conventional methods owing to decreased time consumption, micrometer range resolution, and increased accuracy. As expected, based on our previous studies and OCT-based plant studies, the gradual structural changes of the inner morphology were revealed through the continuously examined cross-sectional patterns via images. Perhaps the most interesting finding from this study is the formation of a circular tissue pattern underneath the epidermis, which is the main initial symptom that cannot be identified through conventional inspection techniques. On the other hand, additionally analyzed axial and lateral intensity profiles, and the tissue boundary patterns based on 3D OCT images revealed the potential advantages of this methodology. Therefore, the proposed OCT technique was capable in recognizing the most important stages of bitter rot disease at an earlier stage within a short acquisition period. In addition to *ex vivo* assessments, the developed experimental procedure can be applied to various *in vivo* plant materials while performing field trials (on field inspections) using a wearable OCT device. This may be an ideal solution for *in vivo* inspections that maintains the biological nature of specimens^39^. Moreover, OCT has the capability to detect fluidic flow and the flow velocity non-destructively. Since OCT has been broadly applied for the detection of blood flow and flow velocity, advancements of the aforementioned methodologies can be applicable for the analysis of plant related fluid dynamics. In order to correlate the bitter rot disease progression with micro fluidic flow dynamics of fruit specimens, assessments of internal plant flow dynamics based quantitative evaluations can be applied to investigate the initial disease formation for further examinations. Thus among the techniques, double correlation OCT based superficial blood vessel spatial distribution detection and highly sensitive Doppler-OCT techniques^48,49^ can play a potential role in investigating aforementioned flow dynamics in the future studies.

## Conclusions

The initial detection of morphological variations in apple specimens caused by bitter rot disease at different stages of infection was demonstrated using 1310 nm SS-OCT by examining specimens at periods over 25 days. Among various conventional postharvest inspection techniques, OCT stands out because of its non-destructive nature and increased spatial resolution. Apart from the methodologies used previously, the fundamental objective of the current study is the pre-identification of initial rotting state in apple specimens through continuously monitored 2D-OCT images, intensity profiles, and circular tissue pattern formations in both enface OCT and boundary detection analysis. The identification of circular tissue patterns generated underneath the epidermal cell layer due to initial rotting is a visually non-identifiable fact that was well-emphasized through the acquired enface representations. This could be considered as one of the major findings of the study, which contributes to the existing knowledge-base as an effective method of identifying apple bitter-rot disease at a prior stage. To the best of our knowledge, these experimental findings have not been applied for bitter-rot identification of plant materials to date. The acquired two-dimensional, three-dimensional visualizations and the intensity fluctuations sufficiently confirmed the desired results by compensating the ultimate limits of the existing techniques. Thus, there is no doubt that OCT has already matured as an imaging technique with its beneficial characteristics of increased sensitivity and extraordinary resolution that will continue to improve, thereby rendering the technique as an important tool in biology, agriculture, and the nanosciences.
